# ^14^C-Cobalamin Absorption from Endogenously Labeled Chicken Eggs Assessed in Humans Using Accelerator Mass Spectrometry

**DOI:** 10.3390/nu11092148

**Published:** 2019-09-08

**Authors:** Marjorie G. Garrod, Heidi A. Rossow, Christopher C. Calvert, Joshua W. Miller, Ralph Green, Bruce A. Buchholz, Lindsay H. Allen

**Affiliations:** 1 USDA, ARS Western Human Nutrition Research Center, Davis, CA 95616, USA; 2 Population Health and Reproduction, University of California, Davis, CA 95616, USA; 3 Department of Nutritional Sciences, Rutgers University, New Brunswick, NJ 08901, USA; 4 Department. of Medical Pathology and Laboratory Medicine, University of California, Davis, Sacramento, CA 95817, USA; 5 Center for Accelerator Mass Spectrometry, Lawrence Livermore National Laboratory, Livermore, CA 94550, USA

**Keywords:** cobalamin, vitamin B12, bioavailability, eggs, endogenous label, human, accelerator mass spectrometry

## Abstract

Traditionally, the bioavailability of vitamin B-12 (B12) from in vivo labeled foods was determined by labeling the vitamin with radiocobalt (^57^Co, ^58^Co or ^60^Co). This required use of penetrating radioactivity and sometimes used higher doses of B12 than the physiological limit of B12 absorption. The aim of this study was to determine the bioavailability and absorbed B12 from chicken eggs endogenously labeled with ^14^C-B12 using accelerator mass spectrometry (AMS). ^14^C-B12 was injected intramuscularly into hens to produce eggs enriched in vivo with the ^14^C labeled vitamin. The eggs, which provided 1.4 to 2.6 μg of B12 (~1.1 kBq) per serving, were scrambled, cooked and fed to 10 human volunteers. Baseline and post-ingestion blood, urine and stool samples were collected over a one-week period and assessed for ^14^C-B12 content using AMS. Bioavailability ranged from 13.2 to 57.7% (mean 30.2 ± 16.4%). Difference among subjects was explained by dose of B12, with percent bioavailability from 2.6 μg only half that from 1.4 μg. The total amount of B12 absorbed was limited to 0.5–0.8 μg (mean 0.55 ± 0.19 μg B12) and was relatively unaffected by the amount consumed. The use of ^14^C-B12 offers the only currently available method for quantifying B12 absorption in humans, including food cobalamin absorption. An egg is confirmed as a good source of B12, supplying approximately 20% of the average adult daily requirement (RDA for adults = 2.4 μg/day).

## 1. Introduction

Access to methods for quantifying the absorption of vitamin B12 (B12) from foods is important in setting dietary requirements for the vitamin, and for assessing food cobalamin malabsorption which can cause B12 depletion and deficiency, especially in the elderly. No methods are currently available for quantifying B12 absorption from foods in humans [1]. The only method for detecting clinical B12 malabsorption, the CobaSorb test, is qualitative and does not provide data on the % of B12 absorbed [2].

The only natural dietary sources of B12 are animal products. However, B12 in animal source foods is bound to protein and its bioavailability from these foods depends on factors such as the complexity of different food protein structures and the body’s ability to release B12 from food proteins in the stomach [3,4]. In the Framingham Study [5], strong associations were observed between plasma B12 concentrations and intake of the vitamin from dairy products, eggs, meat, and seafood, but the data suggested differences in the relative bioavailability of B12 from these sources.

In the past, to determine the bioavailability of B12 from foods animals were injected with or fed radiocobalt (^57^Co, ^58^Co or ^60^Co) labeled B12, which became incorporated into the liver, meat, or eggs of the animal. These endogenously labeled tissues were then fed to humans and the amount of labeled B12 appearing in plasma, urine, and stool was measured to determine bioavailability [6,7,8,9,10,11,12,13,14,15]. Gamma ray counters used to measure radiocobalt isotopes measure the decay of the radiosubstrates rather than the actual amount present in a sample. Consequently, limitations in sensitivity and precision required the use of radiosubstrates with relatively high specific activity and often a dose of labeled B12 an order of magnitude larger than the limit of B12 absorption through the physiologic, intrinsic factor-mediated pathway (i.e., ≈2–3 μg from a single meal) [8,9,16,17].

Accelerator mass spectrometry (AMS), a highly sensitive technology for the detection and quantification of ^14^C-labeled substrates at attomole (10^−18^) concentrations [18], directly counts ^14^C atoms rather than radioactive decay events [18] and is approximately one million-fold more sensitive than scintillation counting. The synthesis of ^14^C-labeled B12 (^14^C-B12) [19] provides a means to utilize AMS technology in B12 bioavailability studies in humans. AMS typically requires samples containing only 0.5 mg of carbon, enabling analysis in microliter-sized biological samples even when the dose consists of a small amount of substrate with low specific activity [20]. Absorption studies can be carried out with minimal radiation exposure for the participant. Finally, the long half-life of ^14^C (5730 y) enables sample collection over a longer period of time, which increases the accuracy of the study [21].

The aim of the study was to determine in humans the bioavailability and absorbed B12 from chicken eggs endogenously labeled with ^14^C-B12 using AMS. The yolk of a single large raw egg contains up to 20% of the Recommended Dietary Allowance (RDA) of 2.4 μg [22]. Hens readily deposit large amounts of labeled B12 in egg yolk when injected with the vitamin [23,24], potentially producing eggs with sufficient ^14^C-B12 label for use in bioavailability and absorption studies.

## 2. Materials and Methods

### 2.1. ^14^C-B12 Synthesis

**Figure 1:**
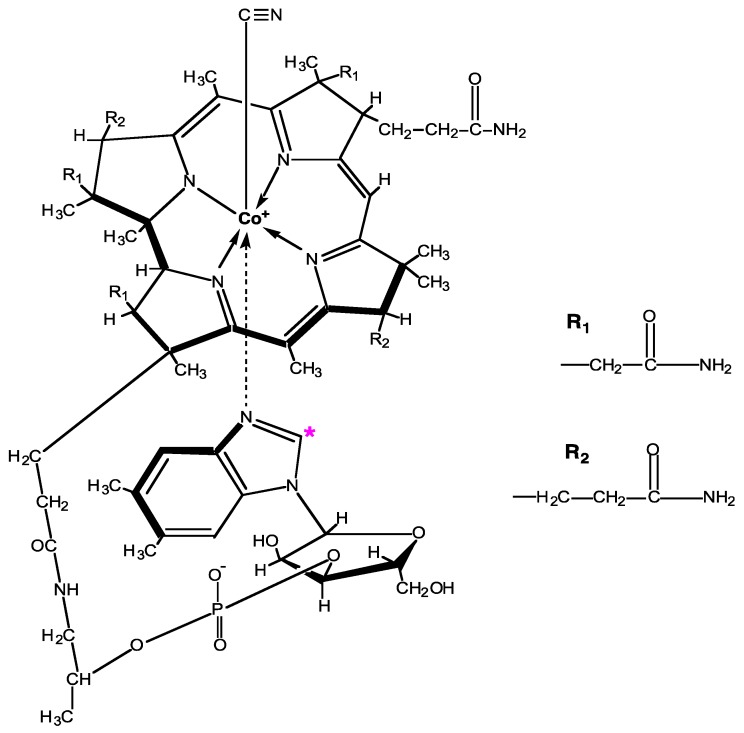
Chemical structure of cyanocobalamin with the ^14^C location indicated by the * in the DMB moiety. Image modified from Green and Miller [27].

^14^C-B12 was synthesized by *S. enterica* (serovar Typhimurium) strain TT24733, genotype *cbiD24::MudJ*. The bacteria were grown aerobically on ethanolamine and incubated with dicyanocobinamide and ^14^C-dimethylbenzimidazole (DMB), which under these growth conditions are dedicated precursors for the biosynthesis of B12 [19]. After a 24 h to 48 h incubation, the newly synthesized ^14^C-B12 was extracted and purified by high performance liquid chromatography (HPLC). The product was confirmed as B12 by ultraviolet-visible spectrophotometry and liquid chromatography-mass spectrometry [19]. The synthesis incorporates a cyanation step to convert all forms of B12 to cyanocobalamin with the ^14^C label located as shown in Figure 1.

### 2.2. Production of Labeled Eggs

All experimental procedures involving the use of animals were conducted in accordance with the Universities Federation for Animal Welfare Handbook on the Care and Management of Laboratory Animals [25] and approved by the Animal Use and Care Committee at the University of California, Davis (#13036). Four White Leghorn laying hens aged 1.5 y were housed in individual laying cages in the University of California, Davis, Avian Science Unit. Temperature was maintained at 24–25 °C. The 24-h light cycle was 20 h light and 4 h dark with light fixtures present on both ceiling and walls. Air exchange was continuous. Hens received a commercial diet for laying hens (Layena, 5.5 µg B12 per pound; Purina Mills, St. Louis, MO, USA) and water ad libitum.

Over a period of 4 d, three individual hens received a total injected dose in the thigh of 13.0, 14.8, or 17.8 kBq (350, 400, or 480 nCi) ^14^C-B12 corresponding to 8.3 µg, 9.4 µg, and 11.3 µg total B12, respectively. The dosing regimen was determined with the aid of a mathematical model for distribution of an intramuscular dose of B12 to other tissues and accumulation in eggs, which is detailed in Appendix B. The fourth hen was used as the control and received no injections. Injections were given at 09:00. Eggs were collected daily from each hen for a month, producing a total of 35 eggs. Eggs produced during the first 16 days were used for the feeding study. Total enrichment of eggs with ^14^C-B12 was determined by counting samples to 1–2% precision in a Liquid Scintillation Counter (Tri-Carb 2500 TR, Model 2700, Packard Instrument Company, Downers Grove, IL, USA). Total B12 in eggs was determined by Covance Laboratories, Inc. (Madison, WI, USA) using turbidometry to compare the growth response of a sample utilizing the bacterium *Lactobacillus delbrueckii* against the growth response to a B12 standard (coefficient of variation: 9.91%) [26].

In the Metabolic Kitchen of the USDA, ARS Western Human Nutrition Research Center, eggs were pooled from each hen, mixed with a hand blender, portioned into servings, cooked in covered plastic containers by immersion in boiling water until the egg reached at least 71 °C, and stored at −20 °C until used in the human bioavailability studies. Total B12 ranged from 1.82 to 2.65 µg/100 g cooked egg. Each serving contained approximately 80 g of egg, 1.1 kBq ^14^C-B12, and 1.44 to 2.65 µg total B12. The specific activity of the servings of cooked eggs varied from 429 to 781 Bq/µg B12 since the ^14^C-B12 content varied amongst the eggs.

### 2.3. Human Subjects, Dosing and Sample Collection

A correlation coefficient power calculation was completed to estimate the population sample size sufficient to detect a significant correlation between individual B12 bioavailability as determined by the fecal excretion method. For this calculation, we made the following assumptions based on previous studies in the literature: A null hypothesis correlation coefficient = 0; an alternative hypothesis correlation coefficient = 0.8; a significance level of *p* = 0.05; a power = 0.8; and a one-sided analysis. Based on these assumptions, the required sample size was calculated to be 8 subjects. A total of 12 subjects started the study and 10 subjects completed it.

**Figure A1:**
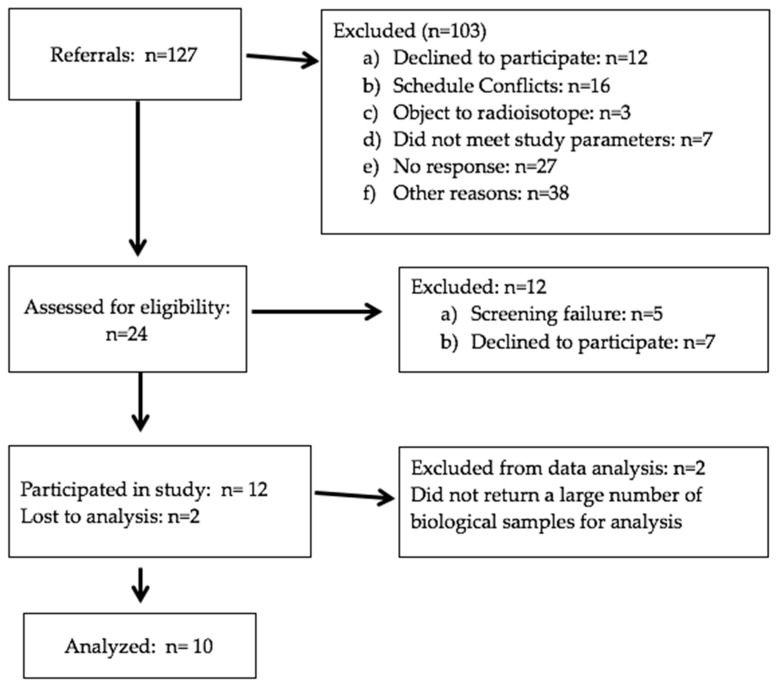
Flowchart of participants’ progress through study.

Subject recruitment and study procedures were approved by the Human Subjects Review Committees at the University of California, Davis (#260218-1) and Lawrence Livermore National Laboratory (LLNL) (#08-103), and written informed consent was obtained from all participants. The study is registered on ClinicalTrials.gov (#NCT01366937). The recruiting, enrollment and study completion flow is described in Appendix A, Figure A1 Inclusion criteria included good overall health based on pre-study survey self-report, adequate B12 status and assumed aborptive capacity as determined by serum B12 assay above the cutoff for deficiency (>148 pmol/L), and the availability to complete the protocol. Exclusion criteria included any chronic health disorder, anemia of any kind, renal insufficiency, and pregnancy or lactation. The radiation dose from ingestion of ^14^C-B12 was calculated to be about 0.030 mSv, equivalent to the exposure during a 6 h commercial airline flight.

Human volunteers were each fed a single serving of the scrambled ^14^C-B12 labeled egg (Table 1) with dry toast and water. Individual doses ranged from 777 to 1158 Bq with total B12 per dose ranging from 1.4 to 2.6 µg depending on the eggs used (Table 1). The adequacy of this dose was determined in a pilot study in which a 2180 Bq dose of crystalline ^14^C-B12 (1.5 µg B12) in water was given to one subject and produced a very strong signal to background ratio in plasma, urine, and stool samples [19]. Upon analysis of the plasma samples of the first subject to complete the study (S2), subsequent doses were adjusted to contain 1.1 kBq of ^14^C. The total dose of B12 varied due the specific activity varying between eggs and consequently servings.

Blood samples were taken at baseline, hourly through 12 h, at 15 h and 24 h, then daily at the time of day the dose was given for an additional 7 d. Twenty-four hour urine and stool samples were collected before dosing followed by collection of all post-dosing voids for 8 d. Meals were controlled to provide no foods of animal origin or containing B12 during the first 24 h. Subjects returned to their normal diet for the remainder of the study.

Stool collections were homogenized in order to obtain representative samples [28]. A stool sample ≤ 350 g was placed in a 3.8 L paint can and an equivalent weight of water added. Fifty grams of 8 mm 316 stainless steel balls was added to the can and the lid sealed with duct tape. The can was shaken on a commercial paint shaker for 30 min, aliquots of 1.5 mL and 50 mL were retained and the remaining stool discarded. The 1.5 mL tube was sent to LLNL for AMS analysis of ^14^C content. The 50 mL sample was retained for possible future analysis. Each sample was processed in a new can with new stainless steel balls to prevent cross-contamination between samples. Aliquots of stool homogenate, urine and plasma were promptly frozen and then shipped overnight to LLNL for graphite preparation and AMS analyses.

### 2.4. Analysis of ^14^C

Samples were prepared as graphite for measurement using standard procedures [29,30]. Since the carbon concentrations can vary widely, carbon concentrations of all urine samples and stool homogenates were measured at LLNL using an Exeter Analytical CE440 carbon analyzer (North Chelmsford, MA, USA) as described [28]. All ^14^C AMS measurements were conducted on the 1-MV National Electrostatics Corporation (Middleton, WI) AMS system at LLNL and normalized to four identically prepared IAEA C-6 isotopic standards [28,29,31]. An isotopic fractionation correction of δ^13^C = −25 per mil was used for all samples and results were reported as described previously [28]. All AMS data are reported as mean ±1 standard deviation.

### 2.5. Calculation of % Bioavailability

Normally, % bioavailability would be determined based on the difference between intake and fecal excretion. However, we found that the percent of the dose of radioactivity excreted in urine is 1–2 orders of magnitude higher than that previously reported when the vitamin was labeled with radioactive cobalt. This phenomenon was first observed by Carkeet et al. [19] who used a similar dose of ^14^C-B12 given in aqueous form. In that study, it was determined that the majority (>99%) of radioactivity in the urine from the aqueous dose was not intact B12, indicating that the majority of ^14^C in the urine may be a degradation product of B12 that was absorbed, and not a bioavailable fraction of the oral dose. Based on this observation % bioavailability was calculated using the sum of both urinary and fecal excretion of ^14^C:

$$\%\;\mathrm{Bioavailability}=\frac{{}^{14}C\;\mathrm{intake}-[{}^{14}C\;\mathrm{excretion}\;\mathrm{in}\;\mathrm{feces}+\mathrm{urine}]}{{}^{14}C\;\mathrm{intake}}\times 100\%.$$

### 2.6. Statistics

Data were examined for normality using the Shapiro–Wilk test. The alpha level was set at 0.05, and a *p*-value > 0.05 is consistent with a normally distributed population. Mean % bioavailability values in subjects receiving lower (1.43–1.66 µg) and higher (2.38–2.65 µg) doses of B12 were compared using Student’s t-test. Both the Shapiro–Wilk test and the Student’s t-test were conducted using Microsoft Excel.

## 3. Results

### 3.1. Appearance of ^14^C Label in Eggs

**Figure 2:**
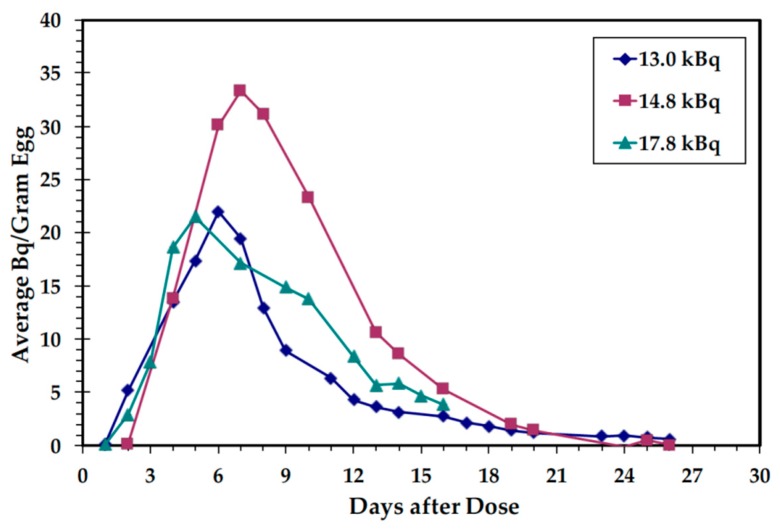
Time course of ^14^C-B12 enrichment of study eggs. The series indicate the ^14^C-B12 doses received by different hens.

**Figure A4:**
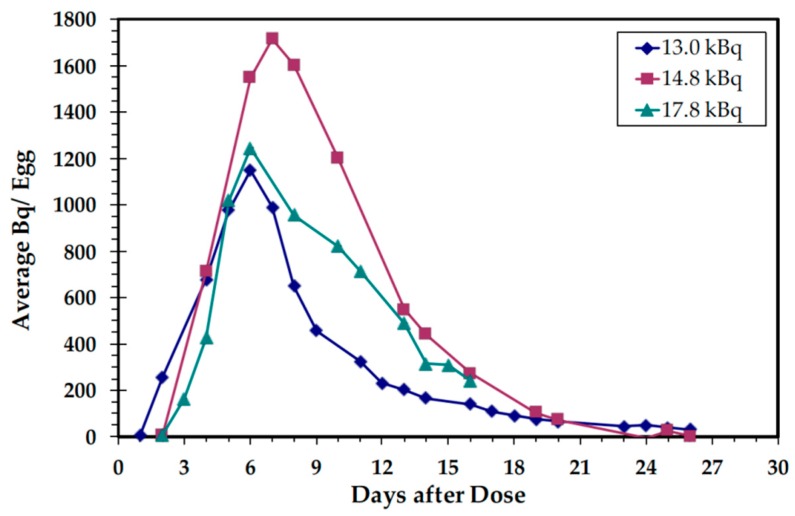
Time course of average ^14^C activity per egg. The series indicate ^14^C B12 doses delivered to different hens.

In the Edwards study [23], peak enrichment was noted in eggs produced 5–7 d after dosing when % dose incorporated ranged from 9.6–10.3%. In the present study, the nCi concentration and range of enrichment in peak eggs was similar to that observed by Edwards [23]. Peak enrichment in each injection protocol was achieved in eggs produced on d 6–8 in all hens and ranged from 7.1 to 12.2% of the total dose given (Figure A4). The initial delay in enrichment is due to the time (approximately 24 h) that the egg remains in the ovary, where the B12 is incorporated into the yolk. Each hen incorporated a different percentage of the total dose into the eggs produced over the initial 16-d time period; 48%, 56%, and 39% for doses of 13.0, 14.8, and 17.8 kBq, respectively. The efficiency of B12 incorporation into the eggs enables endogenous ^14^C-B12 labeling in normal sized servings (Figure 2).

### 3.2. Appearance of ^14^C Label in Plasma, Uurine, and Feces

**Figure 3:**
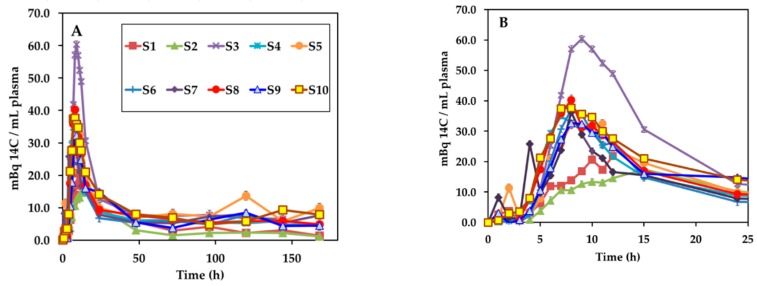
Time course of ^14^C-B12 in plasma after ingestion of in vivo labeled egg (**A**) for the entire study, and (**B**) for the initial 24 h. The error bars for individual data points are 1 SD and generally smaller than the symbol.

^14^C first appeared in plasma approximately 4 h after ingestion of the labeled egg in all volunteers although peak plasma concentrations of ^14^C varied among participants (Figure 3). Most excess plasma ^14^C was cleared by 72 h although a low-level elevation above baseline remained throughout the collection period. Plasma peak ^14^C concentration (Cmax) ranged from 17.4 to 60.3 Bq/L plasma with a mean ± SD peak value of 34.9 ± 12.0 mBq/mL. Plasma area under the curve (AUC) for 0–24 h ranged from 250 to 649 Bq·h/L plasma with a mean AUC of 404 ± 106 Bq·h/L. Plasma area under the curve (AUC) for 0–168 h ranged from 657 to 1785 Bq·h/L plasma with a mean AUC of 1294 ± 327 Bq·h/L. The plasma AUC for all study subjects is given in Table 2.

**Figure 4:**
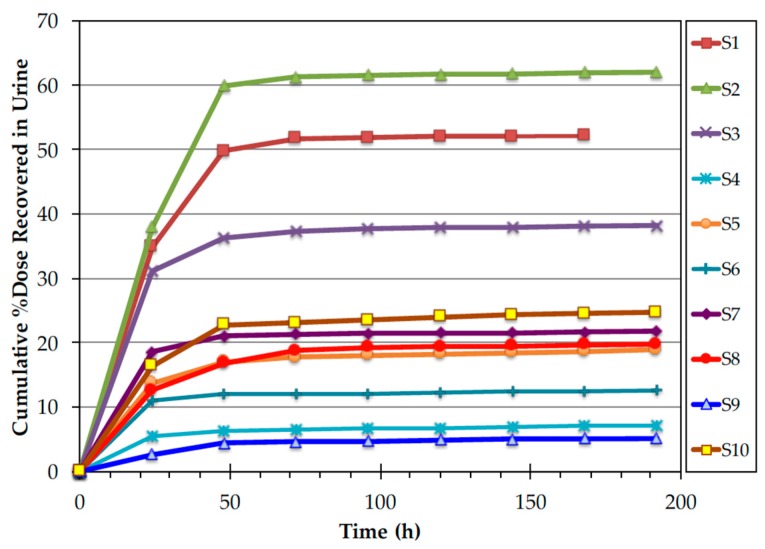
Time course of % dose recovered in urine after ingestion of in vivo labeled egg.

**Figure 5:**
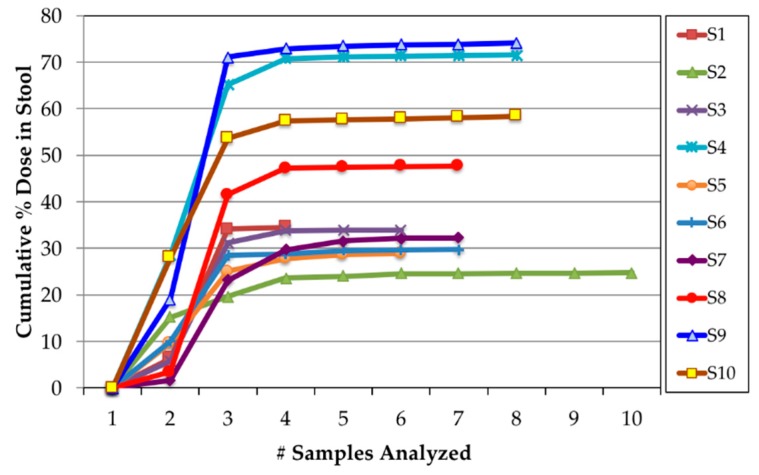
Time course of % dose recovered in stool after ingestion of in vivo labeled egg.

**Figure 6:**
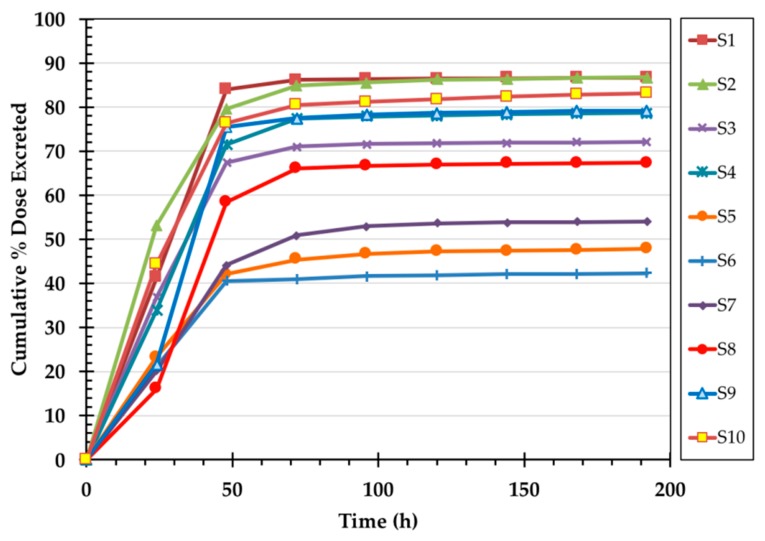
Time course of total % dose excreted after ingestion of in vivo labeled egg.

In all volunteers, the majority of ^14^C label appeared in the first two 24 h urine collections (Figure 4). However, the amount of label recovered in the first 48 h varied widely among the subjects with the cumulative % dose recovered in urine ranging from 5.1 to 62.0% (mean 26.2 ± 18.9%). ^14^C concentrations in urine returned to near background values by the fifth 24 h urine collection (120 h). The majority of fecal ^14^C label appeared in the stool by Day 4. However, as with the urine, the amount of label recovered varied widely among the subjects (Figure 5). ^14^C concentration in stool returned to background concentrations by the fifth 24 h stool collection (120 h). The cumulative % dose recovered in feces ranged from 24.7 to 74.1% with a median 34.2%. The total (urine + stool) excreted ^14^C ranged from 43.2 to 86.7% with a mean of 69.9 ± 16.4% (Figure 6).

### 3.3. Calculation of Bioavailability 

**Figure 7:**
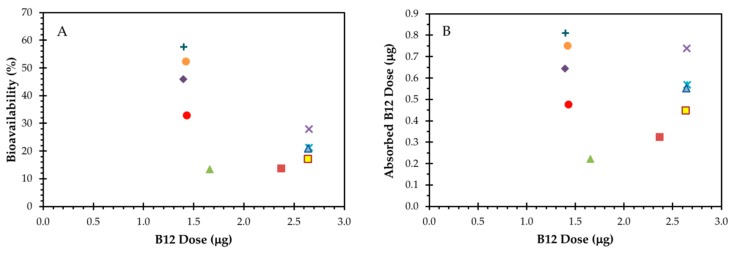

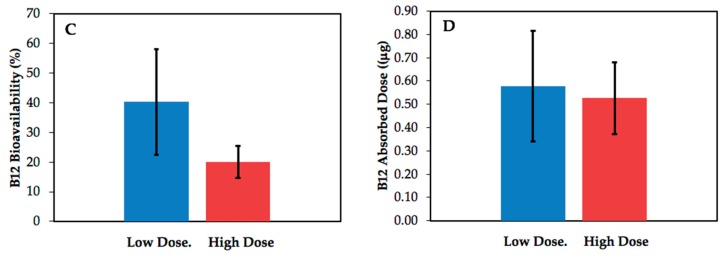
Relationship between bioavailability and total dose of B12. (**A**) Percent bioavailability values for subjects S1–S10. (**B**) The relationship between absorbed dose and total dose of B12. The symbols for the subjects follow the convention of earlier figures. (**C**) Mean % bioavailability values in subjects receiving lower doses of total B12 in the eggs (1.43–1.66 μg, *n* = 5) and in those receiving higher doses of total B12 in the eggs (2.38–2.65 μg; *n* = 5) were compared with Student’s t-test (*p* = 0.04). (**D**) The absorbed dose of B12 was similar between low and high administered doses when compared using Student’s t-test (*p* = 0.69).

The % bioavailability in the 10 volunteers, calculated using the sum of the fecal and urinary ^14^C excretion, ranged from 13.2 to 57.7% with a mean of 30.2 ± 16.4% (*p* = 0.19, Shapiro–Wilk test for normality). Mean % bioavailability values in subjects receiving low doses (1.43–1.66 μg) of total B12 and in subjects receiving high doses (2.38–2.65 μg) of total B12 were significantly different (40.3 ± 17.8% vs. 20.1 ± 5.4%, respectively; *p* = 0.04, Student’s t-test) (Figure 7A,C). The total amount of B12 absorbed and retained was consequently very similar, ≈0.5–0.8 μg for most subjects (mean = 0.55 ± 0.19 μg; *p* = 0.78, Shapiro–Wilk test for normality), across the doses administered (Figure 7B,D).

## 4. Discussion

The use of ^14^C-labeled B12 in this study allowed for a much lower dose of radioactive label for determining bioavailability, compared to past studies using radiolabeled cobalt [8]. For example, Doscherholmen et al. used doses as high as 18.5 kBq of ^57^Co labeled B12 (half-life ~270 d) to determine B12 bioavailability from scrambled eggs in human subjects [6]. In the present study, a 1.11 kBq dose of ^14^C labeled B12 (half-life ~5700 y) was sufficient to raise the concentration of ^14^C far enough above background for accurate detection by AMS in all biological fluids analyzed. In addition, ^57^Co emits penetrating high energy gamma radiation which is far more damaging than the low energy beta radiation emitted by ^14^C.

The bioavailability of B12 from eggs in this study (mean = 30.2%) is similar to the 27.5% reported by Doscherholmen et al. when bioavailability was calculated based on intake minus fecal excretion of ^57^Co-B12 labeled in the corrin ring [6]. Active absorption of B12 from the gastrointestinal tract involves a complex mechanism that requires the gastric production of intrinsic factor (IF). While the capacity of the IF-dependent pathway is limited [16], the binding specificity of IF is very high and requires an intact B12 molecule, limiting the absorption of analogues.

Previous reports indicated that urinary excretion of an oral dose of intact B12 with radiocobalt label is extremely low—far less than 1% of the dose given unless it is followed by a large intramuscular flushing dose as in the Schilling test [16]. In this study, no flushing dose was administered, yet 5 to 62% (mean 25%) of the ^14^C dose was found in urine. In parallel work using an oral dose of ^14^C-B12 in water, the amount of isotope appearing in urine without a flushing dose administered was similar to that observed in this study, and HPLC fractionation verified that the radioactivity in urine was not intact ^14^C-B12 [19]. It is assumed that this is also the case with the same label found in the urine of the current egg study volunteers. Therefore, in calculating bioavailability in this study, it was assumed that the substantial amount of urinary ^14^C detected was not originally absorbed as intact ^14^C-B12.

The mean bioavailability from doses of 1.4 to 2.6 μg of B12 was 30%. A large egg contains approximately 0.6 μg of the vitamin [32], all in the yolk—a lower amount than in our study because injecting the chickens with B12 increased the total amount of B12 in the eggs that we used. Mean bioavailability from 1.44 μg B12 in egg (the equivalent of 2 to 3 normal eggs) in this study was closer to 50% in the 4 subjects given this dose. Because bioavailability decreased with increasing B12 intake our overall estimate of 30% bioavailability may substantially underestimate the actual bioavailability from a single egg.

Most of the wide range in % bioavailability among the 10 human subjects (13.2 to 57.7%) was explained by the fact that different amounts of total B12 were given to the volunteers. The dose of egg was determined based on the experimental decision to supply approximately 1.1 kBq to each subject. While this supplied the same dose of ^14^C-B12 (~1.1 kBq), the amount of total B12 varied between doses because the eggs were derived from different hens, different injection protocols, and contained different amounts of the total vitamin—approximately 1.4, 2.4 and 2.6 μg per dose. This protocol, using different doses of total B12, revealed an inverse relationship between the dose of total B12 and bioavailability, which is consistent with the well-established phenomenon of saturation of the intrinsic factor-cobalamin receptor at higher doses [16]. It was surprising, however, how strong this saturation effect was across the range of relatively low amounts of B12 provided in the single servings of eggs, causing the amount of absorbed vitamin to be very similar across the dose range used.

The Recommended Dietary Allowance for B12 established by the Institute of Medicine is ≈0.5 to 2.4 μg/d across the age range from young children to adults. This recommendation assumes that the usual bioavailability of the vitamin from foods such as meat and fish is 50%, i.e., that the average adult requirement for absorbed B12 is 1.2 μg/d. The current study shows that B12 is absorbed as well from egg as has been reported from fish and meat [1,3,8,13,17]. An egg containing 0.5 μg will therefore provide ≈100% and 20% of the daily recommended intake of the vitamin for children and adults, respectively.

The method for producing eggs endogenously labelled with ^14^C-B12 worked well. The amount of ^14^C-B12 in individual eggs dropped such that eggs laid after Day 16 did not have sufficient label for human servings. Endogenous labels are the best way to trace natural nutrient absorption in food sources. The approach we used works well for small animals and in a ‘tissue’ such as egg that concentrates the nutrient under study. Although not endogenously labeled, it is possible that ^14^C-B12, added to eggs at the point of preparation, could be used to detect food cobalamin malabsorption, following the approach used in the egg yolk cobalamin absorption modification of the Schilling test [1]. Tracing the ^14^C-B12 in plasma, urine and stool was easily accomplished using AMS, but the level of ^14^C is too low for conventional decay counting techniques. Recent developments using cavity ring down spectroscopy [33,34] to measure ^14^C in samples containing a few mg of carbon could be applied to samples such as those generated in absorption studies. Based on the data obtained, it is likely the sampling could have been truncated. Most of the activity was excreted in urine by 48 h and in the stool by Day 4. A limitation of the study was the small sample size (n = 10), but it is noted that the sample size was calculated to be sufficient to provide sufficient power for statistical analysis, and that the calculated mean % bioavailability was very similar to that reported by Doscherholmen et al. [6] using ^57^Co-B12, as discussed above. This provides some confidence that our method using endogenously labeled ^14^C-B12 containing eggs gives a reasonable estimate of % bioavailability of the vitamin from eggs. The study sample also was fairly diverse in age, sex, and BMI, suggesting that the findings may be fairly generalizable to other healthy adults with putatively intact absorptive capacity.

## 5. Conclusions

We confirm that eggs are a good source of B12, and that the experimental approach described here provides a method for quantifying the absorption of the vitamin from in vivo labeled food and for detecting food-bound cobalamin malabsorption, using extremely low doses of radioactivity and low doses of B12. Since urinary and fecal excretion of the label were relatively constant after 48–72 h, the period and frequency of sample collection can be reduced in any future studies. These results also show that more attention needs to be paid to the effect of the amount of total B12 in foods on bioavailability. The latter is especially important for interpreting relationships among intake data, biomarkers of B12 status, and requirements for the vitamin.

## Figures and Tables

**Table 1:** Study subjects and dosing.

Subject	Age (y)	Sex ^1^	BMI ^2^ (kg/m^2^)	Serum	B12	^14^C-B12	Specific
				B12	Dose	Dose	Activity
				(pmol/L)	(μg)	(kBq)	(Bq/μg)
S1	29	F	24.4	331	2.38	1.114	468
S2	43	M	28.2	441	1.66	0.777	468
S3	29	M	24.5	358	2.65	1.159	437
S4	24	F	21.2	323	2.65	1.158	437
S5	53	F	20.8	365	1.43	1.089	761
S6	25	M	23.9	371	1.40	1.083	774
S7	24	M	24.5	306	1.40	1.093	781
S8	26	M	23.8	325	1.44	1.089	756
S9	22	F	35.1	227	2.64	1.131	428
S10	24	M	23.1	386	2.64	1.131	428

^1^ F = female, M = male. ^2^ BMI = Body mass index.

**Table 2:** Plasma absorption and clearance parameters for study subjects S1–S10.

Subject	B12	^14^C-B12	Cmax	Tmax	Plasma	Plasma
	Dose	Dose	Plasma	Plasma	AUC ^1^ 0–24 h	AUC ^1^ 0–168 h
	(μg)	(kBq)	(Bq/L)	(h)	(Bq·h/L)	(Bq·h/L)
S1	2.38	1.114	21.9	12	323	940
S2	1.66	0.777	17.4	15	250	657
S3	2.65	1.159	60.3	9	649	1745
S4	2.65	1.158	34.9	7	386	1241
S5	1.43	1.089	32.5	11	410	1649
S6	1.40	1.083	37.2	8	363	1185
S7	1.40	1.093	36.2	8	357	1291
S8	1.44	1.089	40.2	8	417	1336
S9	2.64	1.131	32.6	8	399	1308
S10	2.64	1.131	37.6	8	483	1585

^1^ AUC = Area under the curve.

**Figure A2:**
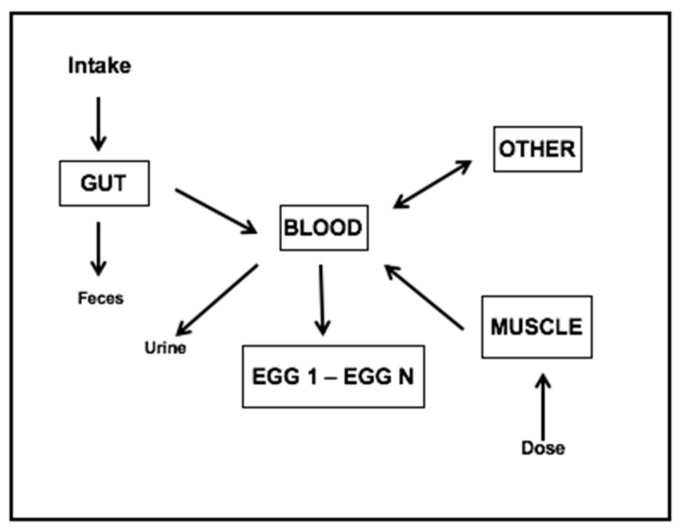
Model diagram representing ^14^C-B12 kinetics in laying hens. ^14^C-B12 is transferred between blood, muscle, gut, all other tissues, and deposited in each egg from egg number 1 to N.

**Figure A3:**
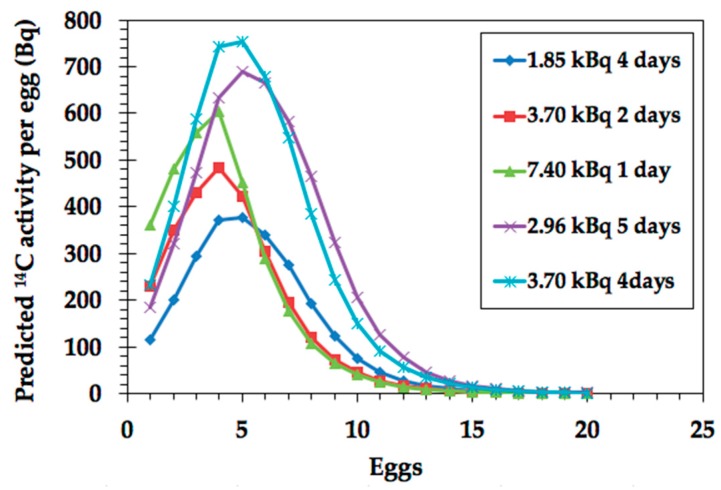
Estimated (model) accumulation of ^14^C B12 in eggs with different dosing schedules.

## Appendix A. Recruitment, Screening and Participation of Subjects

The protocol included a week of participation, so finding a study population willing and able to complete all the blood, urine, and stool collections required a relatively large pool. Of the 12 subjects who started the protocol, 10 completed it. The data for those 10 are used in the reported analyses. A flowchart showing the recruitment, screening and participation of the population is presented in Figure A1.

## Appendix B. Egg Labeling Model

To estimate the ^14^C-B12 dose necessary and to optimize hen dosing schedules, B12 kinetics and transfer into egg were estimated by developing a mathematical model. The model used data from six previously reported dose–response studies [23,24]. Edwards [23] used radioactive B12 while Denton et al. [24] used a dose of the unlabeled vitamin. Differential equations estimated B12 intake and absorption by gut, and transfer of ^14^C-B12 from an intramuscular dose to other tissues, muscle, and accumulation in eggs from blood (Figure A2). Exchanges between state variables were based on mass action kinetics and estimated using Advanced Continuous Simulation Language with a 4th-order Runge-Kutta algorithm for numerical integration of differential equations and an integration interval of 0.01 d (acslXtreme 2007, Aegis Technologies, Huntsville, AL, USA).

The mathematical model was able to replicate experimental results from Denton et. al. [24] and Edwards [23] (Table A1). Observed (literature) and predicted (model) percentage accumulation of B12 dose in all eggs (ODE and PME, respectively), and observed and predicted peak B12 peak eggs (OPE and PPE, respectively) were very close for all experiments (Table A1). Several different ^14^C-B12 doses were simulated to determine which design or dosing schedule would result in the most ^14^C-B12 label accumulation in eggs (Figure A3). The dosing regimen of 100 nCi/d (3.7 kBq/d, 2.36 µg B12/d) for 4 d predicted the maximum percent of dose transferred to eggs. Figure A4 depicts the ^14^C activity per egg for the eggs collected from the three hens dosed with ^14^C-B12.

**Table A1:** Summary of model simulation results for experimental protocols.

Reference	Protocol	Dose ^1^	ODE	PME	OPE	PPE	ODB	PMB
			(%)	(%)	(%)	(%)	(%)	(%)
Edwards [23]	1	0.5 µCi ^57^Co-B12 (IM)	52	52	8	11	35	37
Edwards [23]	2	0.5 µCi ^57^Co-B12 (FED)	33	32	5	4	18	19
Edwards [23]	3	0.072 µCi ^60^Co-B12 (IM)	63	59	11	12	45	41
Edwards [23]	4	0.072 µCi ^60^Co-B12 (FED)	36	37	7	5	30	27
Denton [24]	5	3 µg/d crystal B12 (IM)	33	28	4	4	NA	NA
Denton [24]	6	3 µg/d crystal B12 (FED)	20	15	1.5	1.3	NA	NA

^1^ Abbreviations: IM = intramuscular, ODE = observed data eggs, PME = predicted (model) eggs, OPE = observed peak egg, PPE = predicted peak egg, ODB = observed data body, PMB = predicted model body, NA = not available.
